# Post-material values and brain drain: how value transformation shapes the migration mindset among highly educated South Asians?

**DOI:** 10.3389/fsoc.2026.1775599

**Published:** 2026-05-25

**Authors:** Samitha Udayanga

**Affiliations:** Bremen International Graduate School of Social Sciences, University of Bremen, Bremen, Germany

**Keywords:** brain drain, human capital flight, immigrant integration, latent class, post-material values, South Asians

## Abstract

While economic reasons are predominantly considered as drivers of brain drain in the existing literature, the present study demonstrates that migration decisions are substantially shaped by structural misalignments between individuals’ value orientations and the institutional, cultural, and social environments of their home countries. Drawing on 25 in-depth interviews and a focus group discussion, the study integrates Bourdieu’s theorisations of habitus, field, and taste with Inglehart’s theory of post-material value shift to understand how higher education cultivates durable dispositions oriented toward personal autonomy, quality of life, social justice, and self-actualisation that home-country context structurally fails to accommodate. The analysis reveals that highly educated (potential) migrants constitute a latent social class, defined not by a conscious collective identity but by the objective homology of their capital compositions, shared habituses, and convergent migration trajectories. This latent class formation helps explain not only why migration persists beyond economic motivation, but also why diaspora engagement and transnational knowledge transfer remain limited among these immigrants: when migration resolves a deep incompatibility between habitus and social context rather than a temporary material deficit, the motivational conditions for sustained home-country engagement are fundamentally weakened. Existing return and diaspora policies in South Asia have largely failed in this context, because they often prioritise financial incentives while disregarding the value transformations that originally produced emigration. Therefore, the findings call for a reorientation of policy frameworks toward addressing the structural field conditions, including institutional trust, professional recognition, political openness, and quality-of-life infrastructure, that drive value-motivated human capital flight.

## 1 Introduction

Globally, there were approximately 304 million international migrants in 2024, with European and Central Asian countries hosting a large number of highly educated immigrants (UN Migration, 2024). In OECD countries, around 30–35% of immigrants hold a tertiary degree or higher (OECD, 2024). Favourable social and labour market policies in these countries have attracted highly educated immigrants, particularly from developing regions such as South Asia, Latin America, Northern and Southern Africa (OECD/European Commission, 2023). According to OECD data, India alone accounts for 3.12 million highly educated immigrants (OECD, 2024). Bangladesh, Bhutan, Sri Lanka, Nepal, Afghanistan, Maldives, and Pakistan have also experienced rising emigration of highly educated individuals, making South Asia a key region of origin for highly educated immigrants settling primarily in OECD countries, especially in Europe and North America (Ohnsorge and Xie, 2025; Udayanga, 2024b). Although home countries may benefit from remittances sent by such highly educated individuals, their migration poses a serious setback to socioeconomic development (Stark and Fan, 2007).

Highly educated persons are those who have obtained a tertiary degree or higher and are likely to possess the capabilities necessary for international migration. Higher education qualifications facilitate the development of experiences that serve as basic credentials for entering the global labour market and settling in foreign countries to secure a satisfactory quality of life (Dubey and Mallah, 2015). The transnational global order facilitates their migration, while simultaneously generating high demand for highly educated individuals in many developed countries (Docquier, 2014; Lowell and Findlay, 2001). While not all highly educated individuals develop a migration mindset, those who possess qualifications and skills recognised within international labour markets are generally inclined to migrate when home-country labour markets fail to absorb, reward, or adequately utilise their human capital (Dodani and LaPorte, 2005b; Udayanga, 2024a; Varma and Kapur, 2013).

The decision to migrate among highly educated individuals is multifaceted. One of which is unfavourable socioeconomic conditions in their home countries, which lead them to seek a better quality of life in more prosperous settings (de Haas, 2021; Udayanga, 2024a). Several studies have identified a mismatch between individual qualifications and labour market absorption in the country of origin as a primary cause of brain drain (or human capital flight) (Docquier, 2014; Marsh and Oyelere, 2017; Sen, 1973). Economists, including Acemoglu and James, have emphasised that human capital flight is a key reason why nations often fail to achieve sustained development, mainly in the developing world (Acemoglu and James, 2012). According to the World Bank, human capital flight accounts for 4 per cent of skilled individuals in high-income countries, 10 per cent in middle-income countries, and over 20 per cent in low-income countries, indicating that developing countries are the most severely affected (World Bank, 2023). In response, many developing countries, including South Asian governments have attempted to reform labour market policies to retain talent and encourage the return of highly educated expatriates (Gupta et al., 2024; Hijazi et al., 2024; Kaluarachchi and Jayathilaka, 2024; Skeldon, 2009). However, such measures have had limited success. Each year, a substantial number of highly educated individuals continue to migrate (Gupta et al., 2024; Kaluarachchi and Jayathilaka, 2024; Udayanga, 2024a), suggesting the presence of push factors that go beyond economic and institutional shortcomings. This motivates an examination beyond conventional labour market–focused explanations of brain drain in South Asia, encouraging greater attention to its sociocultural drivers. Therefore, the present study explored how value transformations among individuals function as a driver of migration, with particular focus on the emigration of highly educated individuals.

Highly educated and skilled individuals are crucial for national development, not only as a resource pool for economic growth but also as contributors to broader human development (Cloete et al., 2020; Dodani and LaPorte, 2005; Trinh, 2023). In countries such as Sri Lanka and India, educational reforms have produced a better-educated citizenry capable of advancing national progress (Jayasuriya, 2000; Sharma and Kaldewey, 2025). Bangladesh, Pakistan, Bhutan, Nepal, and the Maldives have also invested in higher education, viewing it as a mandatory investment for human capital development (Asian Development Bank, 2017). Afghan migrants, who are often driven by more urgent existential concerns, also illustrate both the potential and challenges associated with human capital mobility (Mixed Migration Centre, 2023). These individuals are key contributors to innovation, research, health, and education systems, as well as to industrial leadership (Asian Development Bank, 2017). Nevertheless, the continued outflow of such talent from South Asia has resulted in two major losses: the waste of public investment in higher education and the decline of human capital essential for national development (Gibson and McKenzie, 2012; Gupta et al., 2024; Kaluarachchi and Jayathilaka, 2024). Despite the efforts to retain or repatriate them, notable progress remains limited. According to 2024 statistics (World Population Review, 2025), as shown in Table 1, Sri Lanka, Afghanistan, and Bangladesh are disproportionately affected by brain drain, despite a relative decline in human capital flight in India since 2017 (from 6.4 to 4.8).

**Table 1:** Human capital flight index, and country rank.

Country	Brain drain index	Rank
Afghanistan	7.0	25
India	4.8	100
Sri Lanka	7.5	16
Pakistan	5.5	86
Bhutan	5.4	87
Nepal	6.10	59
Maldives	4.9	98
Bangladesh	6.7	37

Source: obtained from world population review data, 2025.

Drawing on Bourdieu’s (1990) conceptualisation of *habitus* and *field* alongside Inglehart’s (2018) conceptualisation of value transformation, this study aims to understand how higher education contributes to transforming values from materialist to post-materialist orientations, which become ingrained in individuals’ dispositions, fostering a migration mindset. While looking into the migration of highly educated individuals from South Asia, who often settled in prosperous countries, the present study makes two key contributions. First, it provides a sociological understanding of human capital flight, with a particular focus on changes in value orientations. Second, it demonstrates that value incongruence between the individual and home-country context is a key driver of migration, particularly in South Asian countries. This mismatch encourages migration to countries that better reflect post material values. This suggests that current policy interventions, which focus solely on economic or labour market solutions, are insufficient. Instead, broader sociopolitical reforms aligned with post-materialist values are necessary to address the ongoing loss of highly educated individuals.

The remainder of this paper is structured as follows: Section 2 reviews the existing literature on human capital flight and theoretical frameworks; Section 3 outlines the methodology; Section 4 presents the empirical findings; and Section 5 discusses the implications and concludes with policy recommendations.

## 2 Literature review and theoretical concerns

### 2.1 Brain drain situation in South Asia

Brain drain refers to the migration of highly educated or skilled individuals from their home countries, which severely disrupts national development (Docquier, 2014; Sen, 1973). Also known as human capital flight, this occurs when skilled and highly educated individuals emigrate due to incompatibilities between their aspirations and the available socioeconomic conditions of their home countries (Marsh and Oyelere, 2017). Human capital flight is often cited as a primary cause of developmental failure, particularly in developing countries (Acemoglu and James, 2012). The drivers of brain drain include both push factors, such as limited opportunities, economic instability, and poor governance, and pull factors, including primarily attractive socioeconomic conditions in highly prosperous countries (Dodani and LaPorte, 2005; Weinar and Klekowski von Koppenfels, 2020). Prosperity, in this context, encompasses more than economic growth alone; it includes dimensions such as low corruption, guaranteed freedom, functioning democracy, safety, security, and overall high quality of life (Fritz and Koch, 2016; O’donnell et al., 2014). As a result, migration generally flows from countries with lower prosperity to those with higher prosperity levels (UN Migration, 2024). As Thompson (2017) highlights, geographical imagination is also a vital process in this regard, through which potential migrants develop mental images of places where they envision a greater quality of life compared to known places where such quality of life is not guaranteed. This also reflects a psychological recalibration through which individuals come to understand their place in home societies relative to their imagined place abroad, ultimately fostering a migrant mindset. This aligns with the aspiration-capability framework, in which de Haas (2021) argues that when aspirations for a higher quality of life are formed, capable individuals are likely to migrate to realise those aspirations.

Among South Asian countries, the Indian diaspora is the largest, exceeding 17 million individuals, many of whom are highly educated (Bhattacharya, 2022; Singh, 2022). Although India has seen a slight decrease in human capital flight since 2017, rising unemployment rates continue to compel skilled individuals to seek opportunities abroad, predominantly in OECD countries (Bhattacharya, 2022; OECD, 2024; World Population Review, 2025). Healthcare professionals, including doctors and nurses, comprise a significant portion of these migrants (Poulain et al., 2023). In comparison, Sri Lanka faces a more severe brain drain challenge, with increasing migration among highly educated individuals driven by economic instability and limited local opportunities (Asian Development Bank, 2017; Kaluarachchi and Jayathilaka, 2024; World Population Review, 2025). Similar trends have emerged in Bangladesh due to recent socio-economic unrest, further intensifying human capital flight (Farzana and Jamil, 2025). Nepal, Pakistan, and the Maldives also experience a substantial brain drain, which impairs their social and economic development. For example, in Pakistan, an unemployment rate of 16 per cent has compelled 37 per cent of highly educated young individuals to migrate (Ahsan, 2024). Mainali (2019) highlights that in Nepal, factors such as inadequate working conditions, substandard education, and widespread corruption, including nepotism and favouritism in recruitment, contribute significantly to the brain drain. The primary driving force behind Afghanistan’s highly educated migration is necessity, mainly because of civil unrest and severe challenges to existence (Vrtana and Rahman Rahmat, 2023). While Bhutan experiences brain drain to a lesser extent, recent studies by Dorji and Hosoe (2025) highlight its negative implications. Across these contexts, economic reasons have been identified as the primary drivers of brain drain, though severity varies by country.

The negative consequences of brain drain are well-known, as it directly undermines social and economic development by weakening critical sectors, including information technology, health, and education (Acemoglu and James, 2012). Several studies have documented these adverse effects, prompting policy initiatives to mitigate human capital flight (Ahsan, 2024; Gupta et al., 2024; Labrianidis and Karampekios, 2022; Singh and Krishna, 2015; Sriskandarajah, 2005). For example, India’s “Viksit Bharat” program emphasises the creation of innovative ecosystems, the improvement of infrastructure, the encouragement of return migration, and the development of education and skills training initiatives (Bortolazzi and Khan, 2023; Hussain, 2015). Nepal, Sri Lanka, and Bangladesh have implemented similar reforms, including wage increases for skilled workers, tax incentives, and labour market adjustments (Asian Development Bank, 2017; Kaluarachchi and Jayathilaka, 2024; Mainali, 2019). Despite these measures, limited success has been achieved, partly due to an inadequate understanding of the sociocultural dimensions that drive migration.

While brain drain is considered disadvantageous for sending countries, brain circulation is regarded as beneficial when highly educated immigrants repatriate or contribute to their home countries through multiple channels, including knowledge circulation, facilitation of migrant networks, and technology transfer. As Saxenian (2005) argued, highly educated migrants do not simply transfer human capital from periphery to core but also create transnational networks through which knowledge, experiences, skills, and resources flow in multiple directions. This is particularly evident among Indian and Pakistani diasporas, whose mediating role in technological and scientific exchange between home and host countries has been well documented (Tripathi and Parth., 2025). However, brain circulation does not function effectively when highly educated migrants limit their engagement to remittance sending without contributing knowledge and experience, which is often observed in the Sri Lankan case (Munas, 2023). Importantly, Wang (2025) observed that even when Western-educated Chinese scholars return to China, anti-Western distinctions constructed by local scholars prevent them from actively participating in effective contributions. They further observed that returnees tend to conceal their acquired cultural capital to assimilate into local scholarly circles. This highlights a critical barrier to cross-border knowledge linkages, as the home culture’s unwillingness to embrace returnees’ dispositions makes them reluctant to contribute actively to national development. Although the present study does not primarily focus on cross-border knowledge linkages or repatriation, it discusses how the sociocultural context in home countries, particularly regarding brain-circulation policies, shapes the development of migration mindsets and individuals’ post-migration considerations of contribution.

### 2.2 Post-material values and habitus

Pierre Bourdieu demonstrates that the *habitus* shapes individuals’ interactions with their social worlds, influencing interests, preferences, and even their physical demeanour (Bourdieu, 1977). For Bourdieu, habitus comprises durable, ingrained dispositions, skills, and modes of thinking and feeling acquired through interacting with social situations, unconsciously (Bourdieu, 2002). Habitus is neither purely the product of individual agency nor entirely determined by social structures, but emerges at their intersection. While structural conditions such as class, education, and institutional arrangements inscribe themselves onto individuals, individuals simultaneously act upon and reproduce these structures through their practices (Bourdieu, 1977). This dialectical relationship means that habitus is not static but gradually reconfigured as individuals are exposed to new social fields and experiences. Importantly, this process operates largely below the level of conscious awareness, as individuals internalise dispositions that feel natural and self-evident rather than socially constructed. In the context of migration, this is particularly relevant, as exposure to higher education can introduce individuals to new fields with distinct values, norms, and ways of perceiving the world, gradually reconfiguring their habitus.

Exposure to new experiences can lead to reconfigurations in individual dispositions, contributing to the formation of latent social classes. This explanation is well aligned with social constructivism (Jung, 2019). Habitus is always in interaction with the *field*, a structured social space in which individual identities are acknowledged and recognised. When the habitus aligns well with the field, individuals gain distinct advantages; conversely, misalignment can lead to disadvantages or dissatisfaction. Moreover, latent classes, or social groups that emerge unconsciously based on shared interests/tastes, demonstrate how social inequalities are reproduced (Bourdieu, 2002). Tastes and interests, which are shaped by habitus, differ between social groups; for example, the middle class typically has different tastes from the working class, which helps maintain social inequalities. Highly educated employed individuals, for example, often develop similar interests and tastes, creating coherent social distinctions from other groups. Although the concept of taste is often associated with cultural preferences such as the arts, it also pertains broadly to life interests and aspirations, which differ across class distinctions. These dynamics shape individuals’ life expectations and aspirations.

Latent social classes are not consciously recognised groupings but rather collectivities that emerge implicitly from shared interests, ingrained dispositions (habitus) shaped by comparable social trajectories, capital compositions, and field exposures. Bourdieu (2002) referred to these as aggregates of individuals who, by virtue of occupying similar positions in social space, develop analogous dispositions, perceptions, and practices without necessarily identifying themselves as a coherent group. Among highly educated individuals, prolonged exposure to educational fields characterised by specific intellectual norms, cosmopolitan orientations, and meritocratic logics produces a convergent habitus that distinguishes them as a latent class, even across national and cultural boundaries. This convergence is not the product of deliberate self-identification but of structurally similar socialisation processes operating through educational institutions, professional environments, and transnational academic fields. The latency of this class formation is precisely what makes it analytically significant: its structuring effects on behaviour, aspiration, and mobility operate independently of actors’ conscious awareness of their class position.

Tastes serve as the primary vehicle through which this latent class formation becomes socially legible and consequential within and across fields. As Bourdieu (2002) demonstrated, tastes are not individual preferences but socially structured dispositions that signal and reproduce class position. Among highly educated individuals, tastes oriented toward intellectual engagement, professional recognition, quality of life, and social openness (aligned closely with post-material values) are not arbitrary but are inscribed through habitus formed within high-capital educational fields. When these individuals encounter a home-country context that fails to recognise or reward such tastes, offering instead limited professional autonomy, misaligned opportunity structures, or restricted social environments, a condition of field-habitus misalignment arises. This misalignment generates subjective dissatisfaction that is experienced personally yet is structurally produced, constituting a key mechanism through which latent class membership translates into migration disposition. The destination-country context, by contrast, offers a social space in which their habitus, tastes, and capital compositions are more readily recognised and valorised, producing the social context-habitus correspondence that Bourdieu identifies as the basis for a sense of belonging and social ease.

In this context, capital composition refers to the accumulated knowledge, skills, competencies, and credentials that individuals acquire through socialisation, which can be converted into social advantages within a given field. Bourdieu distinguishes cultural capital in three forms: embodied cultural capital, as durable dispositions and competencies inscribed in the body and mind through socialisation; objectified cultural capital, as cultural goods; and institutionalised cultural capital, as formally recognised educational credentials (Bourdieu, 1977, 2002). Cultural capital is closely intertwined with habitus, as the accumulation of cultural capital through higher education simultaneously reconfigures individuals’ dispositions, tastes, and modes of perception. In the migration context, higher education serves as a field where individuals accumulate institutionalised cultural capital in the form of academic credentials while also developing embodied cultural capital through exposure to new ideas, values, and ways of engaging with the world.

Bourdieu’s key theorisation offers a key explanation for understanding migration decisions, particularly among highly educated individuals whose habitus aligns with specific latent classes and distinct interests. Therefore, the present study employs the habitus-field framework to examine how highly educated South Asians shape their aspirations and adjust their expectations regarding the quality of life. The congruence or incongruence between habitus and the conditions within a given field thus provides key insights into migration intentions and behaviours. While previous studies have rarely applied habitus-field theory to migration (Erel, 2010), this study highlights how misalignment between aspirations and home-country conditions encourages migration.

Alongside Bourdieu’s conceptualisation, this study employs Inglehart’s theory of post-material values to understand migration trajectories of highly educated individuals (Inglehart and Baker, 2000). Values occupy a central position in sociological accounts of human behaviour and decision-making. Parsons (1971) argued that values function as the normative foundation of social action, providing individuals with shared orientations that guide choices and establish criteria for evaluating desirable ends. In this sense, values are not merely personal preferences but are socially embedded, transmitted through institutions such as family, education, and religion, and internalised as part of individuals’ dispositions. Schwartz (1992) further conceptualises values as trans-situational goals that vary in importance and serve as guiding principles in individuals’ lives, demonstrating that value priorities systematically shape attitudes, preferences, and behavioural intentions across contexts. Inglehart (2018) demonstrates that values are not fixed but undergo transformation in response to changing socioeconomic conditions and formative experiences. This value transformation is particularly relevant to decision-making in the context of migration, as individuals whose value orientations have shifted toward post-materialist priorities may find that the institutional and social structures of their home countries no longer align with their evolved value systems.

According to Inglehart and Appel (1989) and Inglehart (2018), when basic existential needs (economic and physical security) are met, individuals shift their focus towards post-material values, such as self-actualisation, social justice, freedom, autonomy, and overall quality of life (Marčeta et al., 2024). As highly educated individuals secure their material needs, they are likely to prioritise these post-material values. Along with changes in value orientation, individuals’ interests are also reshaped. This value shift, closely associated with economic development and modernisation, is often reinforced by higher education, which typically encourages post-material orientations (Koshy et al., 2023; Van Hiel et al., 2018). Post-material values contrast sharply with survival and traditional values, influencing individuals’ happiness and integration outcomes (Inglehart and Baker, 2000; Sudo, 2022).

While Inglehart’s framework identifies the macro-level conditions under which post-material value orientations emerge, it does not adequately account for why individuals at similar levels of material security differ in the extent to which they embrace and act on post-material values. This is where Bourdieu’s framework provides explanatory depth. Post-material values do not distribute uniformly across a population once material thresholds are crossed; rather, their internalisation as durable dispositions is conditioned by habitus formed through differential field exposures and capital compositions. Highly educated individuals, having been socialised within educational fields that systematically cultivate reflexivity, cosmopolitan orientations, and recognition-based reward structures, are structurally predisposed to incorporate post-material orientations into their habitus. In this sense, Inglehart’s value shift is not a uniform generational phenomenon but is differentially embodied, constituting a class-specific taste that distinguishes the latent class of highly educated individuals from other social groupings sharing similar material conditions.

Inglehart’s theory serves as a macro-structural account of the historical and developmental conditions under which post-material orientations become socially available and legitimate, while Bourdieu’s framework explains the mechanism through which such orientations are selectively embodied, reproduced, and deployed within and across fields. Applied to migration, this means that the post-material aspirations driving highly educated South Asians toward OECD destinations are not merely an intrinsic outcome of having secured material needs, as Inglehart’s framework would suggest in isolation, but are dispositions shaped by habitus formed within transnational educational fields, rendered salient by field-habitus misalignment in home-country contexts, and expressed through migration as a practice of seeking field environments congruent with their internalized value orientations.

## 3 Materials and methods

### 3.1 Design

The present study employed a qualitative research approach, integrating data coming from interviews and focus group discussions (Bryman, 2008). To explore how a transformed habitus aligned with post-materialist values shapes migration motivations among highly educated immigrants, in-depth interviews were conducted with twenty-five South Asian immigrants residing in Sweden, Norway, Germany, Italy, and Australia. These countries were identified as highly prosperous immigrant-receiving contexts (OECD, 2024). Participants were selected based on having migrated as highly educated individuals (those with a tertiary degree or above) and having resided in the host country for more than five years. Additionally, a focus group discussion was conducted with six South Asian immigrants who had settled in Switzerland and Australia. The analysis was informed by a constructivist epistemology, recognising immigrants’ lived experiences as shaped by both individual agency and broader social influences. This approach has several strengths, one of which is its capacity to capture pre-migration existential experiences and to examine how post-migration experiences shape reflections on pre-migration life trajectories. The qualitative approach employed in this study is particularly suited to investigating the sociocultural drivers of brain drain, as it enables the capture of subjective value orientations, experiential narratives, and dispositional shifts. In-depth interviews and focus group discussions allow participants to articulate the interplay between habitus transformation, context-habitus misalignment, and migration motivation in their own terms, preserving the interpretive complexity that is central to understanding value-driven migration. This methodological choice is further justified by the exploratory nature of the study, which seeks to theorise underexamined mechanisms of human capital flight rather than to test pre-specified hypotheses across a representative sample.

### 3.2 Participants and data

Data for the present study were drawn from two qualitative data collection approaches. First, data were collected through in-depth interviews with twenty-five participants, and secondly, this was complemented by a focus group discussion. The initial focus was to understand how highly educated South Asian immigrants living in Sweden, Norway, Germany, and Australia construct their subjective wellbeing and national belonging. However, during the early stages of data analysis, a new concern emerged regarding the incongruence between post-materialist values and the structural and institutional conditions in participants’ countries of origin. Given that existing quantitative data sources often lack information on pre-migration experiences, this concern redirected the qualitative inquiry towards exploring participants’ motivations to migrate to highly prosperous countries, providing insight into the drivers of brain drain. Therefore, the primary focus of data collection was on highly educated individuals, as guided by the theoretical framework. This is one limitation of the present study, as it does not include a comparison with less-educated individuals.

The scope of the qualitative study was limited to South Asian immigrants who had resided in highly prosperous host countries for more than five years. All participants had migrated as highly educated individuals and were between 25 and 65 years old. Participants were recruited using the information power principle, whereby purposive sampling continued until the analytical claims were sufficiently substantiated (Malterud et al., 2021). Recruitment was aimed at representing immigrants from India, Sri Lanka, Nepal, Bangladesh, Pakistan, Bhutan, and the Maldives. Afghan immigrants, whose migration was primarily driven by necessity rather than higher-order aspirations for a better quality of life, were not considered for the in-depth interviews. In addition to individual interviews, a focus group discussion was conducted with six highly educated South Asian immigrants who migrated for higher education and later settled in the host countries. This aimed to understand educational migration pathways and the alignment of values.

Before conducting interviews, the study’s objectives were communicated, and informed consent was obtained. Participants did not consent to the public dissemination of full interview transcripts, except for selected narrative excerpts included in this paper. Interviews were conducted online. The interviews were transcribed verbatim and analysed using reflexive thematic analysis guidelines. Although this method is generally applied at the end of data collection, the present study used it iteratively, without waiting until data collection was complete. Although open coding was initially employed, the coding process was not overly emphasised, as participants’ lived experiences often transcended categorical labels. Therefore, a holistic and reflective analytical approach was adopted, allowing themes to be constructed organically from participants’ narratives. For this purpose, the principles proposed by the reflexive and iterative thematic analysis approach were employed (Udayanga, 2025).

### 3.3 Analytical strategy

Qualitative data were collected from purposively selected participants who met the criteria of length of stay abroad (more than 5 years), educational qualifications (having at least a tertiary degree or above), and origin from South Asia. Participants were primarily recruited through a post shared in social media groups targeting South Asians living overseas. Additionally, some participants joined through referral on the basis of information power (Malterud et al., 2021), as initial participants referred others. There were five data collection stages; each stage included a maximum of five interviews and began data analysis. Until the third stage, all participants came to the interviews after seeing the social media post. All participants were then asked to refer to other potential participants who had been interviewed, and the process began at the fourth stage. This may lead to homophily bias, yet in qualitative studies, snowball sampling inherently consists of this bias (Braun and Clarke, 2019). Importantly, in reflexive sociology, homophily is not a bias; it is a strength in understanding social connectivity and collective experiences in a common group, which is the focus of the present study (Basov, 2020). Given that a Reflexive and Iterative Thematic Analysis approach was employed, both data collection and analysis followed an iterative process. The iterative nature of the study also facilitated the use of sampling by referral. The analysis began after the first five in-depth interviews, and analytical claims were progressively developed throughout the remaining 20 interviews, for a total of 25. Tables 2, 3 provide details of the participants. For the focus group discussion, participants joined by expressing interest in the social media post.

**Table 2:** Participants’ information (in-depth interviews).

No	Gender	Age	Marital status	Home country	Host country
First stage					
1	Male	36	Married	India	Sweden
2	Male	40	Married	India	Norway
3	Male	45	Married	Nepal	Sweden
4	Female	51	Married	Bangladesh	Australia
5	Female	33	Single	Sri Lanka	Germany
Second stage					
6	Female	30	Single	Nepal	Sweden
7	Male	35	Single	India	Australia
8	Male	36	Married	Maldives	Norway
9	Female	37	Single	Sri Lanka	Germany
10	Female	41	Married	Sri Lanka	Germany
Third stage					
11	Male	58	Married	Pakistan	Germany
12	Male	60	Married	Pakistan	Australia
13	Male	57	Married	Maldives	Norway
14	Female	56	Married	Nepal	Sweden
15	Female	57	Married	Nepal	Sweden
Fourth stage					
16	Male	36	Single	India	Italy
17	Female	34	Single	India	Italy
18	Female	33	Single	Sri Lanka	Germany
19	Female	37	Single	Pakistan	Australia
20	Female	31	Single	Bangladesh	Sweden
Fifth stage					
21	Female	36	Married	Bhutan	Norway
22	Male	35	Single	Bhutan	Australia
23	Male	37	Married	Sri Lanka	Sweden
24	Male	46	Married	India	Germany
25	Male	42	Married	Maldives	Italy

**Table 3:** Focus group discussion.

No	Gender	Age	Marital status	Home country	Host country
1	Female	28	Single	India	Australia
2	Female	27	Single	Bangladesh	Australia
3	Male	35	Married	Sri Lanka	Switzerland
4	Male	36	Single	Maldives	Switzerland
5	Male	34	Married	Pakistan	Australia
6	Female	28	Single	Nepal	Switzerland

Qualitative data were analysed iteratively using reflexive thematic analysis. While Braun and Clarke’s (2019) guidelines were followed, they were adapted to suit the specific aims of the present study. Accordingly, data analysis began before data collection was complete and was conducted in six stages. Initially, in-depth interviews were analysed, leading to the development of two analytical claims/themes. Subsequently, the focus group discussions were analysed alongside the earlier interview data. The six-step thematic analysis process (Braun and Clarke, 2019) was applied consistently throughout. To ensure rigour, established quality criteria were prioritised throughout the research process (Refer: Udayanga, 2025).

## 4 Findings

The analysis revealed two main motivations behind participants’ migration to highly developed host countries. The first concerns a widely cited driver of brain drain: the incongruence between individuals’ capabilities and the labour-market absorption mechanisms in their countries of origin. Highly educated individuals often receive inadequate recognition or reward for their qualifications and competencies, undermining their expectations of financial security. The present paper does not primarily focus on describing this structural mismatch, even though it remains a significant existential concern for many participants. Instead, this section provides non-financial motivations underlying the migration decisions of highly educated individuals. Traditional migration theories often rely on the push-and-pull framework, and while the findings of this study align in part with that approach, they also unpack more complex social mechanisms that drive migration among highly educated individuals. Unlike conventional labour migrants, these individuals are often motivated by aspirations for quality of life, autonomy, and personal fulfilment rather than material gains alone. Two key analytical claims were developed: the first describes how post-material values embedded habitus are accumulated, centring on a latent class, and the second describes how habitus-social context misalignment develops a migration mindset that focuses on flourishing in a country where their dispositions are aligned. Although subtle variations exist across national contexts, these differences are not the focus of this paper.

### 4.1 Claim 1: latent class formation and post-material values

Higher education is not merely a means of acquiring skills for the labour market; it also plays a crucial role in shaping social classes. As individuals navigate their life trajectories through the lens of higher education, their dispositions (ways of thinking, perceiving, and acting) tend to shift. This transformation reflects a process of internalising values that differentiate them from those without such educational backgrounds. The acquisition of a tertiary degree or higher often provides access to new social networks and cultural capital, aligning individuals (often unconsciously) with a latent social class. Obtaining higher education and accumulating a range of capabilities motivate individuals to form latent classes whose members share similar characteristics and higher-order aspirations, such as the pursuit of a better quality of life. Along this class alignment, their tastes and interests evolve, often aligning with those of the highly educated, and converging with post-materialist values.

First, the process of transformation of value orientations from material to post-material values among highly educated individuals is observed. Participants in this study described how their experiences of higher education transformed their perspectives and expectations, particularly regarding work, consumption, and quality of life. One respondent (a university lecturer) noted:

This is a problem with the system. Just go to a shop, and you can buy dhal, rice, or anything else that is extremely harmful to health because it is often contaminated with chemicals. People think they are healthy, though. When I discuss these matters with my friends, I realised that even if you have money, prestige, or other resources, you cannot escape these systemic problems. Most of those food items are contaminated with chemicals unsuitable for consumption. Many people simply buy and consume them, but even knowing this, how can I avoid it? Simply, it is not a country that can offer you healthy food.

This signals a subjective shift in the respondent’s value orientation, moving decisively beyond material concerns. The grievance articulated is not poverty or food insecurity in the conventional sense; the respondent explicitly acknowledges the possession of money and prestige, but rather the structural inability of the home-country field to deliver conditions commensurate with a habitus oriented toward health, autonomy, and informed consumption. These are recognisably post-material concerns in Inglehart’s framework: once material subsistence is secured, the inadequacy of the system is experienced not as deprivation but as an affront to a cultivated taste for wellbeing, bodily integrity, and environmental quality. Crucially, the respondent does not experience this dissatisfaction in isolation. The repeated invocation of friends who share the same perception and possess similar resources yet cannot escape systemic inadequacy discloses the social architecture of latent class formation. A collectivity of individuals with comparable capital compositions, educational backgrounds, and post-materially oriented dispositions arrives at structurally identical conclusions through parallel but unreflective processes. Their shared taste for conditions the home-country field structurally cannot provide constitutes the objective basis of their latent class position, even in the absence of any formal collective identity or organised expression of grievance.

A healthcare professional extended this logic from consumption to reproduction, articulating how latent class membership reshapes intergenerational strategies:

Some of my friends sent their children to private campuses because they believed that sending them to public universities is a total waste. I therefore sent my kid to a foundation course in artificial intelligence and data science. He dropped out after Grade 11. Going to school after the Ordinary level is misleading. Many of my friends did the same. I prepared him for future migration.

This illustrates how context-habitus misalignment does not remain confined to individual experience but is projected forward into deliberate intergenerational practice. The respondent’s rejection of public educational institutions is not merely a pragmatic assessment of institutional quality but an expression of taste, a disposition toward critical, future-oriented, and transnationally calibrated educational investment that marks a clear distinction from the orientations of those who remain embedded within the home-country field logic. Higher education here functions not as a credential-acquisition mechanism but as a field within which post-material orientations are cultivated, refined, and transmitted across generations. The reference to friends who made identical decisions once again discloses the latent class dynamic: a network of similarly positioned individuals, sharing analogous dispositions and tastes, independently reproducing convergent strategies without conscious coordination. Migration is thus not an improvised response to crisis, but a planned destination inscribed within the habitus and transmitted as a legitimate aspiration to the next generation. The latent class thus reproduces itself not only through shared present dissatisfaction but through the forward orientation of its members’ practices, preparing children for migration as a naturalised trajectory, in the same way that other class fractions naturalise university attendance or professional succession. The misalignment between their cultivated dispositions and the available field conditions in the home country is thereby experienced not as a temporary disruption but as a structural and enduring incompatibility that migration alone can resolve.

Another participant reflected on her experience as a medical professional:

As doctors, we had to work 24/7. It was simply exhausting. I wanted to travel with my family, yet we never found the time. Obtaining leave was seen as a sin. When I asked for leave, they gave it as if they were giving away a part of their body. Although we earned, it was not a worthy life. This was not just my experience, yet it was similar for many of my colleagues.

This reveals more than individual occupational dissatisfaction; it discloses the structural conditions through which a latent class crystallises around shared experiential knowledge of the incompatibility between context and habitus. The respondent’s repeated invocation of colleagues who share an identical experience (similarly qualified, similarly positioned within the professional field, and similarly dissatisfied) is analytically significant. It indicates that the discontent articulated is not idiosyncratic but is structurally produced across a population of individuals whose habituses, reshaped through advanced professional socialisation, have come to incorporate tastes for work-life balance, temporal autonomy, and experiential fulfilment that the home-country institutional field systematically denies.

Economic security, which the respondent explicitly acknowledges as present, no longer functions as a sufficient condition for satisfaction once the habitus has been reoriented toward post-material value priorities. The institution’s treatment of leave as an exceptional concession rather than a legitimate entitlement is experienced not merely as an administrative grievance but as a fundamental failure of field recognition—a refusal to acknowledge the dispositions, needs, and human dignity of individuals whose habitus demands more than productive output. It is through the accumulation and mutual recognition of such experiences across a network of similarly positioned professionals that a latent class consolidates: not through formal organisation or declared solidarity, but through the quiet convergence of parallel dissatisfactions, shared perceptions of institutional inadequacy, and increasingly aligned orientations toward migration as the only viable resolution to a misalignment that the home-country field shows no capacity to address.

Those still engaged in higher education also anticipated the incongruence between their developing habitus and the social conditions of their home countries, demonstrating that latent class formation and migratory disposition can crystallise well before the completion of formal qualifications or entry into professional fields.

As a graduate student, I contemplated how I could possibly live in this country. I am a queer person, and there was no way of living there. People were extremely toxic, and I never thought of staying—even though I could, of course, serve the country for its betterment.

This is particularly instructive because it locates the emergence of migration disposition within the educational field itself, prior to any encounter with the labour market or professional institutions. The respondent’s engagement with higher education has cultivated a habitus oriented toward diversity, personal freedom, and social justice, values that are not incidentally absent from the home-country context but are suppressed by its prevailing socio-political structures. The context-habitus misalignment is therefore not a post-graduation discovery but an anticipatory recognition, formed during the very process of educational socialisation through which the habitus is being reshaped. This is analytically significant for understanding latent class formation: it demonstrates that the collectivity of value-driven potential migrants is continuously reproduced through the educational field itself, which functions simultaneously as the site of habitus transformation and as the lens through which home-country conditions become legible as inadequate.

Students undergoing this transformation do not simply acquire qualifications; they acquire a restructured perceptual schema through which the home country is experienced as a field incongruent with who they are becoming. Migration is thereby incorporated into the habitus as a naturalised future trajectory, not just a reactive response to post-graduation disappointment, but a disposition formed and consolidated during education, rendering the latent class self-reproducing across successive cohorts of highly educated individuals even before they formally enter the migration process.

**Figure 1:**
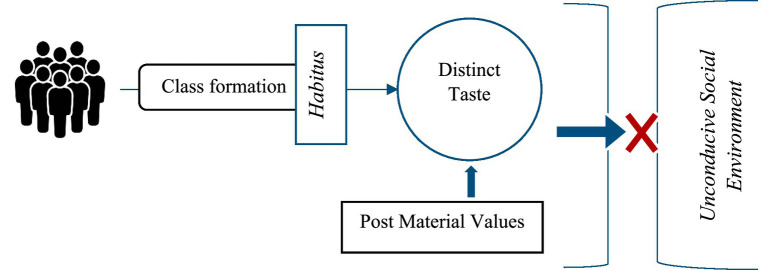
Latent class formation and value incongruence. This figure illustrates the summary of the first analytical claim, developed by the author.

Figure 1 illustrates the process of latent class formation and the alignment of values and tastes that emerge through higher education. It also highlights the disjuncture between these cultivated dispositions and the structural and cultural realities of participants’ home countries. When individuals find that their evolving tastes, preferences, and aspirations, shaped by education and exposure, cannot be meaningfully experienced or expressed in their home contexts, migration becomes a rational and desirable pathway.

As another participant (a worker in a hotel) explained:

I thought, why should I stay there, despite having the capability to fulfil my life goals? I noticed that many of my friends and others had migrated and gone on to lead fulfilling lives. So, my family and I finally decided to migrate to have a fulfilling life.

As this demonstrates, the incongruence between personal tastes and an unsupportive society leads to dissatisfaction and a diminished sense of self-worth. This further exacerbates the mismatch between their capabilities and what they can achieve with those capabilities. This incongruence ultimately leads to migration aimed at highly prosperous countries. Therefore, participants often framed their decision not solely in terms of economic advancement but in relation to the pursuit of a meaningful life that aligns with their post-materialist values. Migration, in this sense, becomes a means of overcoming the incongruence between their internalised habitus and the socio-cultural and political environments of their home countries.

Not all individuals who obtain higher education possess the full range of capabilities required for migration. As the capabilities-aspiration framework suggests, migration is not solely a function of educational attainment but depends on a broader constellation of material resources, social networks, psychological dispositions, and structural opportunities (Carling and Collins, 2018). Those who continue to orient themselves primarily around traditional and survival values are less likely to develop the aspirational dispositions that generate a migration mindset, even when they hold advanced qualifications. This suggests that post-material value orientation functions as a necessary motivational condition for migration among the highly educated, one that must be coupled with a perceived incongruence between those values and the environments available in the home country. Where home-country contexts offer insufficient space for autonomy, self-actualisation, or quality-of-life aspirations, the misalignment between context and habitus intensifies migration disposition. Conversely, where structural barriers constrain the exercise of migration capabilities, as in Afghanistan, where systemic restrictions curtail international mobility regardless of individual aspirations, migration does not follow, even when post-material orientations are present. This confirms that migration among the highly educated is neither intrinsic nor purely economically determined but is mediated by the intersection of value orientation, capability endowment, and structural permission.

A further significant observation is that migration among the highly educated is not confined to professional or qualification-commensurate occupations. Many highly educated migrants enter occupations that do not reflect their educational credentials, a phenomenon indicative of motivations that extend well beyond economic optimisation. This occupational downgrading points toward the primacy of post-material drivers such as personal autonomy, family wellbeing, political freedom, and a broadly conducive social environment, over credential recognition or status maintenance. One healthcare worker holding a postgraduate qualification offered the following account:

“I even have a master’s degree in social research. I am currently working at an elderly care company as a care worker. This does not match my educational qualifications. But if I go to my home country, there is nothing at all. This is far better than going there. I consider my personal autonomy and my family's happiness. That is why I am staying here.

This illustrates a situation in which qualification underutilisation is consciously accepted as a reasonable trade-off against the perceived deficiencies of the home-country field. The respondent does not frame the decision in terms of economic comparison but in terms of autonomy and familial wellbeing, values that are distinctly post-material in Inglehart’s sense and that are rendered into durable dispositions through the habitus formed within transnational educational and professional fields. Overqualification in this context is not experienced as a terminal condition but as a structurally imposed compromise that remains preferable to field-habitus misalignment in the country of origin. This also shows that highly educated migrants often prioritise existential and expressive satisfactions over occupational status when evaluating settlement decisions.

“Before I came here, I saw that many of my colleagues had done the same. Even without a proper job, they had migrated and eventually settled in those countries, which is what they ultimately expected — happiness.” (A clerk)

This narrative does more than illustrate individual motivation; it reveals the social process through which latent class formation occurs among highly educated individuals. The respondent does not refer to formal class identity or collective organisation, yet the narrative reveals a recognisable pattern of shared disposition operating across a network of similarly positioned individuals. Colleagues with comparable educational trajectories, capital compositions, and field exposures arrive independently at convergent decisions — migrating without secured employment, tolerating occupational uncertainty, and orienting their settlement calculus around happiness as a terminal value rather than immediate material gain. It is precisely through this unreflective convergence of aspiration, perception, and practice that a latent class develops. No conscious solidarity is invoked, yet structurally analogous habituses, formed through similar socialisations within high-capital educational fields and experienced as misaligned with home-country field conditions, produce recognisably similar trajectories. In Bourdieu’s (2002) terms, this is a class that exists in practice before it exists in representation: a collectivity constituted through the objective homology of its members’ positions and dispositions rather than through deliberate self-identification. The shared post-material value orientation functions as the affective register of this latent class, simultaneously expressing the habitus formed through educational socialisation and signalling the field conditions that its members collectively seek and recognise as congruent with their internalised dispositions.

Altogether, participants suggested that higher education plays a central role in shaping not only individual capabilities but also worldviews, preferences, and senses of belonging. When these cultivated dispositions are suppressed or misrecognised in the home country, migration becomes a means of aligning one’s habitus with a more congruent social context. This resonates with Thompson’s (2017) discussion of geographical imagination, through which potential migrants develop mental imaginaries of host places where they anticipate flourishing. The same logic applies to value-driven migration, through which highly educated individuals recalibrate their social positioning and develop a migration mindset oriented toward aligning their reconfigured habitus with a more conducive environment.

However, not all highly educated individuals belong to latent classes as described above. Those who continue to prioritise materialist values, centred on economic security, income maximisation, and physical safety, might exhibit substantially different patterns of migration decision-making. For these individuals, migration may remain largely instrumentally driven, oriented toward tangible economic returns and occupational advancement rather than the pursuit of value congruence or ontological security. They might not develop a migration mindset either. This distinction is theoretically significant, as it suggests that the relationship between higher education and migration motivation is not uniform but is mediated by the nature and direction of value transformation. While higher education broadly reconfigures habitus, the extent to which it shifts value orientations from materialist to post-materialist priorities varies across individuals depending on their social origins, institutional experiences, and the cultural capital accumulated prior to and during higher education. Therefore, latent class membership among highly educated potential migrants is not determined by educational attainment alone but by the degree to which post-materialist value orientations have become durably inscribed in individuals’ dispositions, rendering habitus-context misalignment the primary driver of migration rather than material deprivation or economic calculation.

### 4.2 Claim 2: incongruence between habitus and social context

The alignment of personal interests and aspirations with post-materialist values appears to represent a key transformation among highly educated (potential) immigrants in South Asia. When these individual tastes and preferences are reshaped along with post-materialist ideals, they tend to manifest within a latent class framework, as discussed in the first analytical claim. The reconfigured habitus thus functions, reflecting one’s class and cultural capital. The first analytical claim outlined how migration motivations often stem from the misalignment between an individual’s habitus and the socio-institutional conditions of their home country. The second claim builds on that argument by illustrating how post-materialist values develop and become dislocated from the structural and institutional realities of South Asian societies. Generally, it demonstrates that home-country contexts often fail to align with interests rooted in post-materialist values, leading to a latent “migration mindset” among highly educated individuals.

A major focus participants raised concerns about is the tension between equality and conservative cultural expectations. Participants, particularly women, identified traditional socio-cultural norms as barriers to personal and professional growth. Participants described these traditions, embedded within a patriarchal framework, as not only outdated but also actively obstructive to individual agency and emancipation. A school teacher and a software engineer explain:

I remember that very unnecessary things were given extreme prominence. For example, the dress code. As a woman, you had to wear a saree to the workplace. But it had no impact on the actual work. That’s just one example. Even at home, all the care work fell on me. There was no recognition of shared responsibilities between men and women.

We decided not to have children. But this became a serious issue in our community. It wasn’t a problem for us, but for others. It was really invasive. We couldn't make our own decisions because people constantly interfered.

These narratives illustrate a clear clash between the participants’ reconfigured habitus, rooted in values of autonomy, gender equality, and freedom of choice, and the collective social expectations in their home countries. Migration, therefore, emerges not simply as a response to material deprivation but as a pursuit of congruence between self and society—a search for environments that recognise and enable post-materialist life goals. This is more than a conventional “brain drain”; it represents a form of human capital flight rooted in value dissonance.

Another recurrent driver of migration is the aspiration to secure a more supportive educational environment for children. Participants often described their national education systems as rigid, overly standardised, and unaccommodating to creativity and individuality. Therefore, concerns about their own futures and a forward-looking vision for their children shape their motivations for migration. These are associated with post-material values in a reshaped habitus.

One of my main goals was to integrate my children into a universal education system. Restricting them to one country was no longer relevant. They need to learn how the world is evolving and adapt accordingly. That just was not possible in our home country.

My son was good at chess and dancing. But in Sri Lanka, the teachers wanted to confine him to schoolwork. That’s not how you identify or cultivate talent.

Our time is over. Why should we sacrifice our children’s future? They need to go further than we did. Otherwise, they’ll end up like frogs in a well.

These narratives highlight a distinctive aspect of post-materialist orientation: a long-term investment in personal development, creativity, and the realisation of potential. Parents who subscribe to these values view global mobility as a necessary strategy to provide their children with access to environments where such aspirations are institutionally supported.

Participants also described their home societies as “toxic” spaces fundamentally misaligned with their values and aspirations. While not broadly generalisable, participants often characterised their home countries as toxic in terms of how they are treated and how their social position is received. This perceived toxicity encompassed interpersonal envy, lack of trust, and systemic barriers to self-actualisation. The dissonance between their evolved habitus and the prevailing social ethos is experienced as psychologically and emotionally taxing.

From a Bourdieusian perspective (2002), this toxicity can be understood as a manifestation of hysteresis, the condition arising when a reconfigured habitus becomes structurally misaligned with the social context in which it operates. As higher education reshapes individuals’ dispositions, value orientations, and modes of perception, the home country may no longer provide the conditions necessary for the habitus to function effectively, generating a persistent sense of misfit and discomfort. This misalignment is not merely cultural but operates at a deeper psychological level, as individuals whose habitus has been transformed through education experience the home country’s norms, social hierarchies, and reward structures as fundamentally incongruent with their evolved self. The perceived toxicity thus reflects the emotional and psychological costs of inhabiting a field that misrecognises or actively devalues one’s reconfigured dispositions and cultural capital.

People were extremely toxic. When someone is successful, others get jealous and try to bring them down. People are more aware of others but not themselves!

Today I saw a Facebook post in [home country] where someone's new car was set on fire, out of jealousy. Living there, you expect trouble. You can't be yourself. People are just waiting to sabotage others.

These reflections illustrate more than cultural discomfort; they signal a profound incompatibility between participants’ transformed habitus and the social field in which they remain embedded. The envy and sabotage described are symptomatic of a field that penalises distinction and suppresses the very dispositions that higher education cultivates, such as autonomy, achievement orientation, and post-materialist value expression. Migration, then, becomes not merely a pragmatic response to structural constraints but an emotionally driven imperative to exit a field experienced as hostile to one’s evolved sense of self, and to reinvest intellectual and emotional capital in environments where such capital is recognised, valued, and reciprocated.

Another concern relates to dissatisfaction with traditional work ethics in the home country. Participants expressed frustration with environments in which overwork is valorised, and leisure is considered a privilege rather than a right. For highly educated individuals with secure incomes, the emphasis shifts towards achieving a better work-life balance and greater overall life satisfaction.

Having leisure is important. But I had to live for work. My life became completely derailed, and I didn’t get anything out of it—not even a satisfying vacation.

Vacation was a forbidden word. You couldn’t even ask for it. So why should I keep working there?

In Sri Lanka, taking leave is a privilege, not a right! It’s written in regulations. Ridiculous! But in many European countries, it’s the opposite. Those outdated laws serve only toxic systems.

The lived experiences of participants exemplify how post-materialist values reframe the meaning of work. The participants are not rejecting work, but rejecting its instrumentalisation. They desire conditions where their professional contributions are balanced by the right to rest, autonomy, and a dignified life, an ethos that stands in contrast to the prevailing norms in many South Asian contexts. The presented narratives demonstrate that more than just economic gain motivates migration among highly educated South Asians. It is propelled by a deep disjunction between post-materialist values and traditional socio-cultural and institutional settings. The misalignment between habitus and environment generates not only dissatisfaction but a moral and emotional imperative to seek out alternative social fields where one’s values, skills, and life aspirations are nurtured rather than suppressed. This is not merely a case of “brain drain,” but a broader human capital flight fuelled by cultural incongruence. These migrants seek not only better jobs, but better lives, encapsulating safer societies, more equitable institutions, meaningful autonomy, and a more holistic sense of wellbeing.

A core concern across participants’ narratives is the aspiration toward a flourishing life, one that extends beyond material security to encompass dignity, recognition, autonomy, and holistic wellbeing. When the home country social context fails to accommodate these aspirations, the misalignment between the reconfigured habitus and the prevailing social context does not remain a passive source of dissatisfaction but actively generates a migration mindset oriented toward environments perceived as more conducive to self-realisation. Participants consistently articulated a forward-looking imagination of life elsewhere, where their evolved dispositions, values, and capabilities would not merely be tolerated but institutionally and socially supported.

For participants, the forecasted wellbeing imaginaries were not abstract but were concretely anchored in comparative assessments of social conditions, institutional frameworks, and cultural norms between home and prospective host countries. The home country, experienced through the lens of a reconfigured habitus, appeared as a field that foreclosed rather than enabled the realisation of post-materialist aspirations, while imagined host environments were constructed as spaces of possibility where one’s transformed sense of self could be expressed and valued.

Giddens (1991) theorisation of ontological security is particularly instructive here. Ontological security refers to the sense of continuity, stability, and trust in the social and material environment that underpins individuals’ overall wellbeing and capacity to function with confidence in everyday life. For Giddens, this security is not merely a psychological state but is sustained through routine, predictable social interactions and institutional arrangements that affirm individuals’ sense of self and place in the world. When habitus-context misalignment intensifies, the conditions necessary for ontological security are greatly reduced. Participants’ narratives of toxicity, misrecognition, and suppressed autonomy reflect this decline, as the home social field becomes an environment that destabilises rather than affirms their evolved sense of self. The inability to routinely enact one’s reconfigured values and dispositions without social sanction or institutional obstruction generates a pervasive sense of existential unease, further consolidating the migration mindset as individuals seek environments where ontological security can be restored and sustained.

Critically, this process is not reducible to rational calibrations alone but is deeply embedded in the emotional and psychological dimensions of habitus-context misalignment. As participants’ dispositions evolved through higher education, the home field’s inability to provide the social and institutional conditions necessary for habitus-field congruence rendered everyday life untenable. The cumulative experience of misrecognition, whether through suppressed autonomy, devalued cultural capital, or structural barriers to self-actualisation, gradually consolidated into a migration mindset framed not as escape but as a pursuit of congruence between one’s evolved self and the social environment. Migration, in this sense, is understood by participants as a deliberate reinvestment of their intellectual, emotional, and cultural capital in fields where ontological security is perceived as structurally available and the conditions for a flourishing life are institutionally sustained.

## 5 Discussion and policy implications

Human capital flight has become a pressing concern in South Asia, with the loss of highly educated individuals significantly hindering the region’s development. Importantly, this is not solely driven by financial incentives (Ahsan, 2024; Kaluarachchi and Jayathilaka, 2024; Mainali, 2019). As the present study reveals, migration among highly educated South Asians is also strongly influenced by a mismatch between their value orientations and the socio-cultural conditions of their home countries. Although the present study is limited to South Asian countries, the results are comparable primarily to those of countries in the Global South.

The findings extend the existing knowledge by showing that the migration decisions of highly educated South Asians are motivated by a desire for value alignment, particularly the aspiration to live in societies that support post-materialist values. These values, including personal freedom, quality of life, social justice, and self-actualisation, are often difficult to fulfil in the more conservative or survival-oriented contexts of many South Asian societies. In accordance with Inglehart’s value typology (Inglehart, 2018), participants perceived their home countries as lacking institutional support for post-materialist values, creating a dissonance with their reshaped habitus. As a result, many sought to migrate to highly prosperous countries (predominantly to OECD countries) where such values are widely acknowledged and institutionalised. Therefore, having migratory aspirations is not sufficient to develop migration decisions, as suggested by de Haas (2021); they require value concerns to set the mindset for migrating. However, the present findings do not imply that highly educated individuals migrate solely due to a misalignment between value orientations and country conditions. As some studies show, even highly educated individuals may migrate when their existential needs are unmet, as in the case of Afghanistan (Barlas, 2022; Vrtana and Rahman Rahmat, 2023). Nevertheless, migration driven by a desire for a better quality of life and happiness is often influenced by incongruence between personal value orientations and conditions in their home countries. These observations are well-aligned with Thompson’s argument.

While previous explanations for brain drain in South Asia have focused on economic or employment-related factors (Docquier et al., 2007), the present study highlights the neglected social and psychological dimensions of migration. Highly educated individuals are not merely seeking better jobs, but environments in which their aspirations and lifestyle preferences are socially and institutionally supported. The aspiration for a better quality of life extends far beyond the pursuit of mere economic stability. As the capacities of highly educated individuals can more readily be translated into favourable labour market outcomes, they often prioritise improving their quality of life, an ambition closely linked to post-materialist value concerns. At the same time, several studies indicate that highly educated migrants often encounter significant barriers to labour market access in more developed host countries, which may reduce their anticipated post-migration happiness (Lowell and Findlay, 2001; Nowok et al., 2013; Salt, 2004; Udayanga, 2024a). Nevertheless, the present study suggests that, despite such challenges, high-calibre immigrants tend to remain in host societies largely because they anticipate greater value congruence. Post-migration experiences show that South Asian immigrants gradually achieve higher levels of happiness and integration, especially in highly prosperous countries. This contrasts with several previous findings that highlight a decline in immigrants’ happiness (Diehl and Trittler, 2024; Schaeffer and Kas, 2024; Yaman et al., 2022). These results suggest that not all immigrants experience country conditions in the same way; rather, such experiences are moderated by individual dispositions.

The education and capabilities of highly educated individuals are more effectively utilised and rewarded in more prosperous countries, leading to sustained wellbeing and a diminished intention to return, as described by OECD (2023). The present findings confirm that return intentions are minimal among participants, primarily due to the lack of a favourable environment in their home countries, particularly in terms of values, lifestyle, and institutional support. Although recent studies suggest a trend of return migration from countries like Canada and the United States (Lo et al., 2019; Pinkattitude, 2023; Udayanga, 2024b), this study finds little evidence of return to South Asia. Instead, many of these individuals opt to relocate within Europe. This points to a critical gap in current South Asian policy frameworks. While efforts have been made to reverse the brain drain, such as offering higher salaries, vehicle permits, or favourable loan schemes, these policies have had minimal effect. The core issue remains unaddressed: the mismatch between migrants’ value orientations and the institutional and cultural conditions in their countries of origin.

The “toxicity” as invoked by participants warrants careful interpretive caution, as it is an emic term articulating a subjective perception shaped by the respondents’ own dispositional framework rather than an objective characterisation of the social environment. The sense of toxicity experienced by participants is analytically understood here as an affective expression of habitus-field misalignment: when a habitus oriented toward post-material values such as autonomy, diversity, and social openness encounters a field structured around incompatible norms and institutional logics, the resulting incongruence is experienced and narrated as an intolerable social atmosphere. This perception is not idiosyncratic but operates at the level of the latent class, in which individuals sharing analogous habituses and capital compositions tend to develop convergent perceptual schemas through which home-country field conditions are similarly appraised as restrictive, hostile, or incongruent with their cultivated dispositions. It should be noted, however, that this finding is not generalisable beyond the study’s participants, and the present analysis does not claim that home-country environments are objectively toxic; rather, it demonstrates that the recurring invocation of toxicity across participants consistently indexes the same underlying condition of habitus-field incompatibility, making it a analytically meaningful signal of the value misalignment that drives migration disposition among this population.

These observations are well-aligned with Thompson’s (2017) argument that migration decisions are not exclusively driven by economic rationality but are deeply underpinned by emotionally charged geographical imaginations, through which individuals construct mental imaginaries of places perceived as more conducive to flourishing. Participants’ articulations of life elsewhere were not merely aspirational abstractions but were affectively loaded comparative assessments, in which the home country’s perceived toxicity, institutional misrecognition, and structural foreclosure of post-materialist aspirations were imaginatively contrasted with host environments constructed as spaces of possibility, recognition, and ontological security. This emotional dimension is theoretically significant, as it demonstrates that the migration mindset consolidates not solely through rational cost–benefit deliberation but through an imaginative and affective process in which the evolved habitus actively participates.

Moreover, the findings of this study carry important implications for debates on transnational knowledge circulation and diaspora engagement, even though neither constitutes an explicit analytical theme here. Main analytical claims offer a structural explanation for why diaspora engagement and cross-border knowledge transfer remain limited among this population, a gap that the existing literature has acknowledged but insufficiently theorised. When migration is driven not by temporary economic calculation but by a deep and durable incompatibility between habitus and the social context, the motivational conditions for sustained engagement with home-country institutions are fundamentally undermined. Migrants who have left precisely because the home-country field failed to recognise, reward, or accommodate their cultivated dispositions, tastes, and post-material orientations carry with them a habitus that is oriented away from that field rather than in productive tension with it. Unlike migrants whose departure is instrumentally motivated and potentially reversible, those whose migration resolves a structural value misalignment are unlikely to re-engage with the very field conditions they experienced as incongruent. Furthermore, the latent class character of this migration reinforces its insularity from diaspora mobilisation: because the collectivity is constituted through shared disposition rather than conscious solidarity, it generates no organisational infrastructure through which diaspora engagement policies might activate collective contribution. Gamlen (2008) observed that diaspora engagement mechanisms often fail due to political incoherence in sending countries, but the present study suggests an equally significant demand-side constraint, that the habitus of value-driven migrants is structurally misaligned not only with home-country labour markets but with the affective and normative conditions that would make sustained transnational engagement meaningful or desirable to them. This finding calls for a reorientation in diaspora policy frameworks: rather than designing engagement mechanisms premised on migrants’ presumed loyalty or latent developmental obligation, sending-country institutions must first address the field conditions, such as institutional trust, political openness, professional recognition, and quality-of-life infrastructure, that originally produced the misalignment driving emigration.

The migration of highly educated individuals from South Asia also carries significant implications for social transformation and the advancement of the Sustainable Development Goals (SDGs), particularly in relation to the institutional capacities of sending states (Piper, 2017). Human capital flight systematically depletes the professional, scientific, and intellectual resources upon which states depend to deliver quality education, healthcare, technological innovation, and effective governance, all of which are foundational to SDG targets in health, education, reduced inequalities, and sustainable economic growth. While diaspora remittances contribute modestly to poverty reduction, they cannot substitute for the embedded institutional knowledge, professional expertise, and civic participation that highly educated migrants would otherwise contribute to strengthening state capacities at home. Conversely, when diaspora engagement conditions are met, particularly where institutional trust and political openness are present, highly educated migrants possess the potential to function as agents of social transformation, transferring internationally acquired knowledge, practices, and networks back into home-country institutions and thereby reinforcing rather than depleting state developmental capacities (Piper, 2017). The present findings, however, suggest that value misalignment and habitus-field incompatibility might constrain this potential, as migrants whose departure resolves a deep structural incongruence are unlikely to re-engage with the institutional environments they experienced as inadequate, ultimately widening the gap between SDG aspirations and the human capital resources available to pursue them.

Importantly, certain limitations of the study should be acknowledged. The qualitative analysis primarily aims to highlight the significant role of value orientations among highly skilled individuals. However, this paper does not address variations that may arise from cultural differences across South Asian countries. Despite these differences, common patterns of value change are observed across the region. Although the sample recruited for qualitative data analysis is limited in size, participant selection was guided by the principle of information power, suggesting that adding more participants would not meaningfully expand the analytical claims. Individual characteristics such as marital status, gender, and age are vital determinants of migration experiences, yet the present study did not focus on this heterogeneity in presenting its analytical claims. Some studies mistakenly apply quantitative robustness criteria to qualitative research; however, in Big Q qualitative studies, such practices risk introducing bias and undermining the validity of findings. Therefore, this study adheres to Braun and Clarke (2025) criteria for evaluating qualitative research quality. In addition, it must be noted that the differentiation between materialist and post-materialist migration trajectories among highly educated individuals remains underdeveloped in the present study, and constitutes a productive direction for future empirical and theoretical inquiry.

This study is the first to explicitly identify value orientation as a central driver of human capital flight among highly educated South Asians. Policymaking in the region must therefore go beyond labour market reforms. Policies must acknowledge and accommodate changing values, especially regarding work–life balance, freedom of expression, and quality of life. For example, reforms in leave policies, flexible work arrangements, and supportive institutional environments for personal growth and gender equality are urgently needed. Without addressing these fundamental issues, the trend of human capital flight is likely to intensify. Attracting already emigrated highly educated individuals will remain a formidable challenge, given the perceived toxic socio-political environments in many South Asian contexts. The deeply ingrained habitus-context misalignment that originally drove emigration is not easily reversed through financial incentives alone, as the underlying value incongruence persists irrespective of material inducements.

Nevertheless, institutional reforms that recognise and adapt to evolving value orientations could at least slow this trend and improve retention prospects. Such reforms would necessarily extend beyond economic policy to encompass the structural field conditions that post-materialist individuals prioritise, including strengthening institutional trust, advancing gender equity, protecting professional autonomy, expanding political freedoms, and investing in quality-of-life infrastructure. Importantly, creating environments where cultural capital accumulated through higher education is genuinely recognised and valued, rather than misrecognised or subordinated to locally entrenched hierarchies, is essential to restoring the ontological security that highly educated individuals seek. Retention, in this sense, is fundamentally a question of field transformation rather than remuneration. If these structural dimensions are ignored, the present findings signal a heightened and largely unrecognised risk to the long-term development potential of South Asian countries, as the continuous outflow of post-materialist human capital represents not merely an economic loss but an erosion of the innovative, reform-oriented dispositions that are indispensable to sustainable social and institutional development.

To conclude, the focus of the present study is to understand how value transformations at the individual level function as a migration driver among highly educated South Asians, moving beyond the predominantly economic explanations that dominate existing brain drain literature. The findings demonstrate that higher education does not merely enhance human capital in instrumental terms but fundamentally reconfigures individuals’ habitus, cultivating post-materialist value orientations. When these evolved dispositions encounter home-country social fields characterised by patriarchal norms, institutional misrecognition, toxic social dynamics, and structural barriers to self-realisation, a profound habitus-context misalignment emerges that consolidates into a migration mindset oriented toward environments where flourishing is perceived as structurally attainable. Highly educated potential migrants were further shown to constitute a latent social class, unified not by conscious collective identity but by the objective homology of their reconfigured dispositions, capital compositions, and convergent migration trajectories. This latent class formation also helps explain the limited diaspora engagement and transnational knowledge transfer observed among this population, as migration driven by value incongruence rather than temporary material deficit fundamentally weakens the motivational basis for sustained home-country contribution. Collectively, these findings reframe brain drain not as a straightforward economic phenomenon but as a value-driven human capital flight rooted in deep structural incongruence between evolved individual dispositions and the social, cultural, and institutional conditions of home societies. Addressing this incongruence, rather than merely competing on material incentives, constitutes the central policy imperative for South Asian countries seeking to retain and re-engage their highly educated populations.

## Data Availability

The original contributions presented in the study are included in the article/supplementary material, further inquiries can be directed to the corresponding author.
